# In-phasic cytosolic-nuclear Ca^2+^ rhythms in suprachiasmatic nucleus neurons

**DOI:** 10.3389/fnins.2023.1323565

**Published:** 2023-12-20

**Authors:** Sota Hiro, Kenta Kobayashi, Tomomi Nemoto, Ryosuke Enoki

**Affiliations:** ^1^Biophotonics Research Group, Exploratory Research Center on Life and Living Systems (ExCELLS), National Institutes of Natural Sciences, Okazaki, Aichi, Japan; ^2^Division of Biophotonics, National Institute for Physiological Sciences, National Institutes of Natural Sciences, Okazaki, Aichi, Japan; ^3^School of Life Science, The Graduate University for Advanced Studies (SOKENDAI), Okazaki, Aichi, Japan; ^4^Section of Viral Vector Development, National Institute for Physiological Sciences, Okazaki, Aichi, Japan

**Keywords:** circadian clock, intracellular Ca^2+^, SCN, imaging, nucleus, organelle

## Abstract

The suprachiasmatic nucleus (SCN) of the hypothalamus is the master circadian clock in mammals. SCN neurons exhibit circadian Ca^2+^ rhythms in the cytosol, which is thought to act as a messenger linking the transcriptional/translational feedback loop (TTFL) and physiological activities. Transcriptional regulation occurs in the nucleus in the TTFL model, and Ca^2+^-dependent kinase regulates the clock gene transcription. However, the Ca^2+^ regulatory mechanisms between cytosol and nucleus as well as the ionic origin of Ca^2+^ rhythms remain unclear. In the present study, we monitored circadian-timescale Ca^2+^ dynamics in the nucleus and cytosol of SCN neurons at the single-cell and network levels. We observed robust nuclear Ca^2+^ rhythm in the same phase as the cytosolic rhythm in single SCN neurons and entire regions. Neuronal firing inhibition reduced the amplitude of both nuclear and cytosolic Ca^2+^ rhythms, whereas blocking of Ca^2+^ release from the endoplasmic reticulum (ER) via ryanodine and inositol 1,4,5-trisphosphate (IP_3_) receptors had a minor effect on either Ca^2+^ rhythms. We conclude that the in-phasic circadian Ca^2+^ rhythms in the cytosol and nucleus are mainly driven by Ca^2+^ influx from the extracellular space, likely through the nuclear pore. It also raises the possibility that nuclear Ca^2+^ rhythms directly regulate transcription *in situ.*

## 1 Introduction

Almost all living organisms on Earth are regulated by a circadian clock, which anticipates environmental changes for their own survival. In mammals, the master circadian pacemaker is located in the suprachiasmatic nucleus (SCN) of the hypothalamus in the brain (Moore and Eichler, 1972; Stephan and Zucker, 1972), which controls various physiological functions (e.g., sleep–wakefulness cycles, hormone secretion) and animal behavior (Welsh et al., 2010; Mohawk et al., 2012). Rodent SCN is composed of approximately 20,000 neurons in the bilateral side of the hypothalamus, which express a wide range of neuropeptides and their receptors (Van den Pol, 1980; Wen et al., 2020) and constitute a hierarchical multi-oscillator system (Welsh et al., 2010). In individual SCN neurons, circadian rhythms are observed in clock gene transcription (Yoo et al., 2004), neuronal firing (Welsh et al., 1995), hormone secretion (e.g., vasoactive intestinal peptide) (Shinohara et al., 1995), and intracellular Ca^2+^ concentration (Colwell, 2000; Ikeda et al., 2003; Enoki et al., 2012a). The molecular background of the circadian rhythms of the biological rhythms is based on the transcriptional/translational feedback loop (TTFL), which is composed of clock genes and their products. In the main loop of this model, circadian locomotor output cycles kaput (CLOCK) and brain and muscle ARNT-like1 (BMAL1), as positive regulators, activate the transcription of the *Period* (*Per*) and *Cryptochrome* (*Cry*) genes via the E-boxes. The expressed CRYs and PERs form their own complex and translocate into the nucleus. Then, they act as negative regulators to repress their own expression and generate rhythms (Mohawk et al., 2012; Takahashi, 2017).

The PER2 expression rhythms were abolished when intracellular Ca^2+^ was chelated in SCN tissue culture (Lundkvist et al., 2005). Furthermore, the behavioral rhythm in *Drosophila* was prolonged when parvalbumin, a Ca^2+^-binding protein, was specifically expressed in clock neurons, suggesting that Ca^2+^ is an important component of the TTFL (Harrisingh et al., 2007). As circadian Ca^2+^ rhythms are attenuated by *Bmal1* knockdown (Ikeda and Ikeda, 2014) and *Cry1/2* knockout (Enoki et al., 2017b), Ca^2+^ and TTFL are likely to be mutually coupled. A hypothesized model of the molecular mechanism of TTFL regulation by the intracellular Ca^2+^ is the phosphorylation of CLOCK through the Ca^2+^/calmodulin-dependent protein kinase (CaMK) II (Kon et al., 2014). In the signaling pathway of light entrainment, glutamate input from the retina triggers Ca^2+^ signaling via NMDA receptors to activate CaMKII and initiate PER transcription via CREB-CRE signaling (Golombek and Rosenstein, 2010). TTFL may regulate Ca^2+^ influx by controlling membrane potential via circadian regulation of Na^+^ and K^+^ conductance (Hastings et al., 2018); however, tetrodotoxin (TTX), a Na^+^ channel blocker, does not suspend cytosolic circadian Ca^2+^ rhythms. It has been reported that Ca^2+^ release from ryanodine receptors on the endoplasmic reticulum (ER) contributes to cytosolic Ca^2+^ rhythm, but the results are either supportive (Ikeda et al., 2003; Plante et al., 2021), negative (Noguchi et al., 2017), or mixed (Aguilar-Roblero et al., 2016).

Despite decades of research on circadian Ca^2+^ rhythms showing the importance of Ca^2+^ in the TTLF model, the ionic origin of circadian Ca^2+^ rhythms remains to be elucidated. Ikeda et al. (2003) and Wood et al. (2001) previously examined nuclear and cytosolic Ca^2+^ dynamics in mouse SCN and in genetically modified tobacco seedlings, and they detected the Ca^2+^ rhythms in the cytosol but not in the nucleus. In general, the nuclear envelope has a continuous structure with the ER, and ryanodine and inositol 1,4,5-trisphosphate (IP_3_) receptors are expressed on the inner membrane of the nucleus (Bootman et al., 2009; Secondo et al., 2020). Based on these observations, it can be hypothesized that nuclear Ca^2+^ is regulated independently of the cytosol. However, it is difficult to assume the independence of nuclear Ca^2+^ on a circadian timescale as the nucleus has nuclear pore complexes through which molecules smaller than ∼40 kDa can passively diffuse (Lin and Hoelz, 2019) and the diffusion rate of Ca^2+^ ions in cells is orders of magnitude faster than the circadian timescale (Villarruel et al., 2021). To re-evaluate the mechanism of nuclear Ca^2+^ regulation in the SCN neuron on a circadian timescale, we performed dual-color Ca^2+^ imaging in the nucleus and cytosol using highly sensitive and genetically encoded Ca^2+^ sensors, GCaMP6s (Chen et al., 2013) and jRGECO1a (Dana et al., 2016). To further investigate the regulatory mechanisms of nuclear Ca^2+^, we administered drugs that inhibit Ca^2+^ influx via the ER and neuronal firing and also obtained results indicating a regulatory mechanism for nuclear and cytosolic Ca^2+^ concentration in the SCN neurons.

## 2 Materials and methods

### 2.1 Animals

Adult female mice with newborn pups were purchased from an animal breeder (Japan SLC, Inc., Hamamatsu, Japan). The animals were housed and provided with food and water *ad libitum* under controlled conditions (temperature, 22°C ± 2°C; humidity, 40% ± 20%; 12-h light/12-h dark cycle, with lights on from 8:00 AM to 8:00 PM). Light intensity was adjusted to approximately 100–200 lx at the cage surface. The animals were fed commercial chow (Labo MR Standard; Nosan Corporation, Yokohama, Japan) and tap water. All animal care and experimental procedures were approved by the Institutional Animal Care and Use Committee of the National Institutes of Natural Sciences and performed according to the National Institute for Physiological Sciences guidelines (approval no. Nos.22A044 and 23A080).

### 2.2 SCN slice culture

The brains of neonate mice (4–6 days old, both male and female) were rapidly removed and dipped in an ice-cold balanced salt solution comprising (in mM) 87 NaCl, 2.5 KCl, 8 MgCl_2_, 0.5 CaCl_2_, 1.2 NaH_2_PO_4_, 26 NaHCO_3_, 25 glucose, 10 HEPES, and 75 sucrose. A 200-μm coronal brain slice containing the mid-rostro-caudal region of the SCN was prepared using a vibratome (VT 1200; Leica Microsystems GmbH, Wetzlar, Germany). The bilateral SCNs were cut out from the slice using a surgical knife and explanted onto a culture membrane (Millicell CM; pore size, 0.4 μ m; Millipore; Merck KGaA, Darmstadt, Germany) in a 35-mm Petri dish containing 1.0-mL DMEM (Invitrogen; Thermo Fisher Scientific Inc.) and 5% FBS (Sigma-Aldrich; Merck KGaA, Darmstadt, Germany).

### 2.3 Gene transfer into SCN neurons

The pAAV-hSyn-NLS-GCaMP6s-WPRE plasmid was constructed by VectorBuilder Inc. The AAV vectors were produced by a procedure as described previously (Sano et al., 2020). AAV1-Syn1-nes-jRGECO1a was purchased from Addgene (Dana et al., 2016). AAV aliquots (0.8–1.0 μL) were inoculated onto the surface of the SCN slices on days 3–4 of culture. The infected slice was cultured for a further 10–14 days before imaging. The titer of all AAV was over 1.0 × 10^13^ genome copies/mL.

### 2.4 Dual-color recording of Ca^2+^ dynamics

For confocal recording, 3–4 days before the recordings, membranes with cultured SCN slices were cut out, flipped over, and transferred to glass-based dishes (3971–035 IWAKI; AGC TECHNO GLASS Co., Ltd., Yoshida, Japan), which were coated with collagen (Cellmatrix Type 1-C, Nitta Gelatin Inc., Yao, Japan). For wide-field recording, culture membrane inserts with cultured SCN slices were transferred to glass-based dishes. Then, the dishes were filled with DMEM (180–200 μL for confocal, 1.2 mL for wide-field) containing 5% FBS and sealed with O_2_-permeable filters (membrane kit, High Sens; YSI Inc.). The specimen was observed using a time-lapse imaging system as previously described (Enoki et al., 2012b). The system consisted of a Nipkow spinning disk confocal unit (X-Light; CrestOptics S.p.A., Roma, Italy), a sCMOS camera Neo (2,560 × 2,160 pixels, Andor Technology, Oxford Instruments, Belfast, UK), an EM-CCD camera Evolve (512 × 512 pixels, Teledyne Photometrics) or an EM-CCD camera iXon3 (1,024 × 1,024 pixels, Andor Technology), a TIXHB box incubator (Tokai Hit., Co, Ltd., Fujinomiya, Japan), and a Ti-E inverted microscope (Nikon Corporation, Tokyo, Japan). Confocal images were acquired with a 20× objective (NA0.75 PlanApo, Nikon Corporation) at 0.975-μm or 0.8-μm resolution. Wide-field images were acquired with a 10× objective (NA0.45 PlanApo, Nikon Corporation) at 2.6-μm resolution. For acquisition to GCaMP6s fluorescence, the specimen was illuminated with LED light (Light Engine; Lumencor) at cyan light (475/28 nm) and fluorescence was visualized with 495-nm dichroic mirrors and 520/35-nm emission filters. For jRGECO1a, 542/27-nm excitation light, 593-nm dichroic mirrors, and 630/92-nm emission filters were used. All experiments were performed at 36.5°C and 5% CO_2_. The intensity of the excitation light and the exposure time of the sCMOS and EM-CCD camera were adjusted to obtain optimal images of each sample.

### 2.5 Pharmacological experiment

Wide-field time-lapse imaging was performed for 4 days, the medium was switched to DMEM containing 0.1% DMSO or inhibitors [10 μM dantrolene (Supelco; Merck KGaA, Darmstadt, Germany), 100 μM ryanodine (Sigma-Aldrich), 1 μM TTX (Abcam plc., Cambridge, UK), 10 μM Xestospongin C (Abcam plc.)], and imaging was continued for another 4 days.

### 2.6 Evaluation of the expression patterns of Ca^2+^ probes

The SCN slices expressing NLS-GCaMP6s or NES-jRGECO1a were fixed with 4% paraformaldehyde in 0.1 M PBS for 60 min at room temperature. The slices were mounted on a glass bottom dish with ProLong™ Diamond Antifade Mountant with DAPI (Invitrogen). Fluorescence was visualized using a confocal microscope (TCS SP8, Leica Microsystems GmbH) equipped with 63 × objective lens (NA 1.20, HC PL APO, Leica Microsystems GmbH) with the following excitation/emission spectra: DAPI (405 nm/410–483 nm), NLS-GCaMP6s (488 nm/493–592 nm), and NES-jRGECO1a (587 nm/592–779 nm).

### 2.7 Data analysis and statistics

For image acquisition, MetaMorph (Molecular Devices, LLC) and LasX (Leica Microsystems GmbH) were used. Fiji (Wayne Rasband, National Institutes of Health) was used to analyze the obtained image data. The neuronal condition was monitored by bright-field imaging at the same time as fluorescence imaging, and the data in which significant cell death, the slice shape change, and the network desynchronization occurred during the time-lapse measurement were excluded from the analysis as they were assumed to be cytotoxic due to light irradiation. To analyze the circadian Ca^2+^ rhythms in the SCN network, we used a rhythm-fitting program (ImageJ Plugin). Time-lapse images were subjected to 7 × 7 or 8 × 8 binning for analyzing phase differences and 2 × 2 binning for other analysis, noise-filtered (median filter, rad = 1.0 pixel), and converted from 16- to 8-bit images. For each pixel data, the following cosine functions were fitted using the least-squares method in 48-h intervals.


$$Y_{p}\left(t_{i}\right)=M_{p}\left(t_{i}\right)+A_{p}\left(t_{i}\right)\cos\left(W_{p}\left(t_{i}\right)\left(t-t_{i}\right)+\Phi_{p}\left(t_{i}\right)\right)$$

where Y_p_ (ti) denotes the signal intensity at time t_i_ (h); M_p_ (t_i_), average fluorescence; Ap (t_i_), amplitude; W_p_ (t_i_), angular velocity; and Φ_p_ (t_i_), phase. To evaluate each fitting rate, an index of the goodness of fit of the fitted values to the measured data was calculated and an uncorrelated test was conducted at a significance level of *p* < 0.001 for making the phase-difference map and *p* < 0.00001 for another analysis, such as calculating the percentages of amplitude and trough changes. Only pixels that were rejected were extracted to generate images. In the phase map, phase differences are indicated by pseudo colors, and pixels rejected by the fitting test are shown in white. The acrophase map of the entire SCN region was normalized to zero and displayed.

Statistical analyses were conducted using Microsoft Excel and RStudio (RStudio, PBC). Paired or unpaired *t*-tests were used when comparing two dependent and independent group means.

## 3 Results

### 3.1 Detection of the nuclear and cytosolic Ca^2+^ dynamics in the SCN neurons

**Figure 1:**
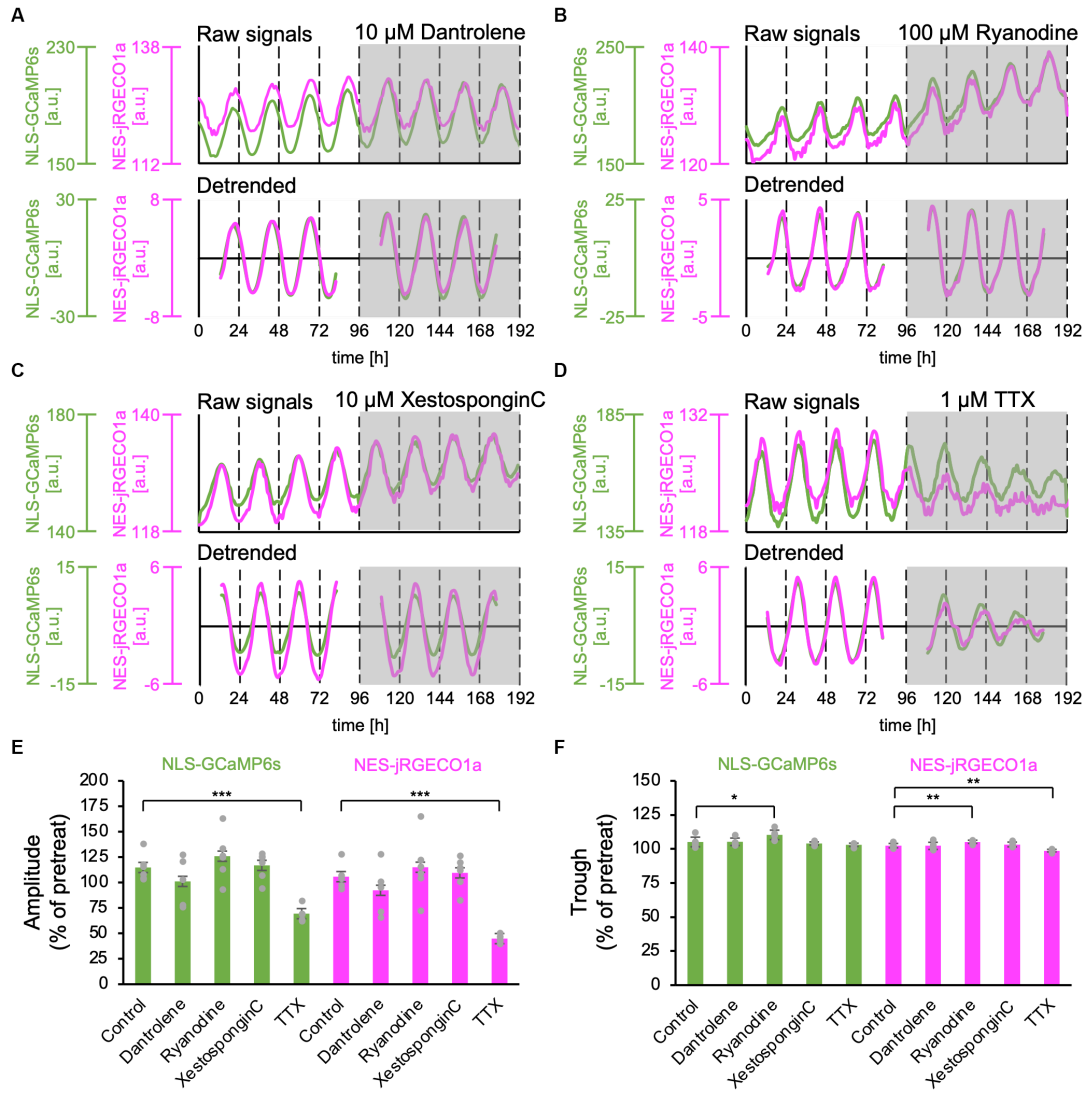
Expression Patterns of the Ca^2+^ Probes in the SCN Slices. **(A–B)** Expression patterns of the probes of NLS-GCaMP6s **(A)** and NES-jRGECO1a **(B)** in the whole SCN (left) and in dorsal and ventral SCN subregions (right). ROIs are indicated by yellow squares in the SCN images. Images are NLS-GCaMP6s or NES-jRGECO1a (left), DAPI (middle), and the merged one (right), respectively. 3 V, third ventricle; OC, optic chiasm.

To reveal the circadian-timescale Ca^2+^ dynamics in the nucleus and cytosol in the SCN neurons at the network and single-cell level, we expressed spectrally distinct and genetically encoded Ca^2+^ probes, GCaMP6s (green) and jRGECO1a (red), in the cultured SCN slices from mice. Nuclear localization signal-tagged GCaMP6s (NLS-GCaMP6s) and nuclear export signal-tagged jRGECO1a (NES-jRGECO1a) were expressed in the SCN neurons in slices using (AAV) transfection under the control of the neuron-specific promoter, human synapsin I. The expressions of NLS-GCaMP6s and NES-jRGECO1a were colocalized or exclusively localized with the nuclear marker (DAPI) at the single-cell level, and both expressions were widely detectable in the entire area, including the dorsal and ventral SCN subregions (Figures 1A,B). These results ensure the origin of the optical signals in the subsequent experiments.

**Figure 2:**
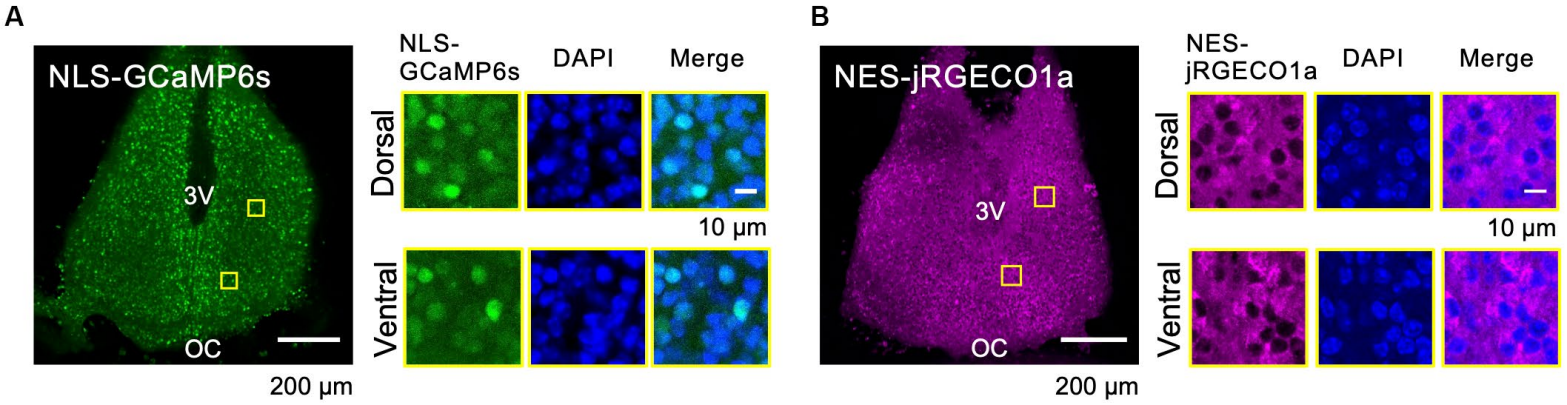
Dual-color imaging of the Ca^2+^ Dynamics in the nucleus and cytosol of SCN Neurons. **(A)** Expression patterns of NLS-GCaMP6s (upper) and NES-jRGECO1a (lower) in the SCN. 3 V, third ventricle; OC, optic chiasm. **(B)** Two-hourly montage images of two representative individual SCN neurons, as indicated by arrowheads in panel **(A)**. The leftmost images show the edge of single SCN neuron. **(C)** Raw traces of the Ca^2+^ dynamics of the nucleus (green) and the cytosol (magenta) of two representative SCN neurons. **(D)** Acrophase maps of the Ca^2+^ rhythms of the nucleus (left) and the cytosol (right). The mean acrophase of the entire SCN region was normalized to zero. Color bars indicate the relative time of day (hours). **(E)** Phase-difference map between the nuclear and cytosolic Ca^2+^ rhythms. **(F)** Histogram of the pixel distribution of the phase-difference maps (normalized to the total pixels). The frequency (y-axis) represents the value relative to the total number of pixels. Individual SCN data are represented by different colors (*n* = 5 slices).

Next, we performed time-lapse confocal Ca^2+^ imaging in the SCN slices expressing NLS-GCaMP6s and NES-jRGECO1a over a period of several days (Figure 2; Supplementary Video 1). We detected circadian Ca^2+^ rhythms in the cytosol and nucleus in entire SCN subregions (Figure 2B). The nuclear and cytosolic Ca^2+^ rhythms were in-phasic at the single-cell level in the dorsal and ventral SCN neurons (Figure 2C). Then, we constructed and compared acrophase maps (peak phase maps) to quantitatively analyze the topological patterns of the nuclear and cytosolic Ca^2+^ rhythms in the SCN network. We found that the spatial patterns of nuclear and cytosolic Ca^2+^ rhythms were almost identical in the entire SCN, with the dorsal subregion phase-leading the ventral subregion (Figure 2D). The phase-difference map (difference of two acrophase maps) confirmed that the two rhythms were in-phasic in the entire SCN regions (Figure 2E). This observation was held true for all the SCN slices examined (−0.19 ± 0.18 h, *n* = 5 slices) (Figure 2F). We further evaluated the phase difference of the two Ca^2+^ rhythms in the dorsal and ventral SCN subregions, normalized to the mean phase of the entire SCN, and found that the phase of nuclear Ca^2+^ rhythm was −1.35 ± 0.77 h in the dorsal and 1.30 ± 1.01 h in the ventral subregions and that of cytosolic Ca^2+^ rhythm was −1.16 ± 0.41 h in the dorsal and 1.22 ± 1.19 h in the ventral subregions, respectively (Supplementary Figure S1C). Significant differences were observed between the dorsal and ventral subregions in both nuclear and cytosolic Ca^2+^ rhythms (*p* = 0.0013 in the nucleus, *p* = 0.0012 in the cytosol) but not between the nuclear and cytosolic Ca^2+^ rhythms within subregions (*p* = 0.26 in the dorsal region, *p* = 0.33 in the ventral region). Single-cell analysis further confirmed our observation that the cytosolic and nuclear Ca^2+^ rhythms were in-phase in individual SCN neurons (0.08 ± 0.33 h, *n* = 30 cells in 5 slices) (Supplementary Figure S1D).

### 3.2 Ionic origin of the nuclear and cytosolic Ca^2+^ rhythms

Ca^2+^ release from the ER has been reported to contribute to the Ca^2+^ rhythm in the cytosol (Ikeda et al., 2003; Plante et al., 2021). Type 2 ryanodine receptors have been reported to be expressed in the SCN (Díaz-Muñoz et al., 1999). Furthermore, it has been demonstrated that the ER and nuclear envelope are structurally interconnected, and the Ca^2+^ release channels, the ryanodine and IP_3_ receptor, are present in the inner membrane of the nucleus (Bootman et al., 2009; Secondo et al., 2020). Thus, it is plausible that nuclear Ca^2+^ may be amplified by Ca^2+^ release from the nuclear envelope as internal stores. To test this hypothesis, we applied inhibitors for ryanodine and IP_3_ receptors, major Ca^2+^ release channels in ER, and examined the nuclear and cytosolic Ca^2+^ rhythms. Dantrolene, a type 1 and 2 ryanodine receptor antagonist (Oo et al., 2015), had no significant effect on the amplitude and trough level of both nuclear and cytosolic Ca^2+^ rhythms (amplitude: 101% ± 21% in the nucleus, 92% ± 22% in the cytosol; trough level: 105% ± 3% in the nucleus and 102% ± 3% in the cytosol) (*n* = 5 slices). To further confirm this observation, we applied another ryanodine receptor antagonist, a high concentration of ryanodine (100 μM), in the SCN slices. High ryanodine concentrations bind to the receptor sites and subsequently cause a decrease in Ca^2+^ conductance (Thomas and Williams, 2012). We found that the amplitude and trough of both nuclear and cytosolic Ca^2+^ rhythms were slightly but significantly increased (amplitude: 126% ± 21% in the nucleus, 115% ± 27% in the cytosol; trough: 110% ± 3% in the nucleus and 105% ± 2% in the cytosol) (*n* = 6 slices). These results indicate that ryanodine receptor-mediated Ca^2+^ release is not a major determinant of Ca^2+^ rhythm generation but rather contributes to the control of baseline levels of Ca^2+^ concentration in SCN neurons.

**Figure 3:**
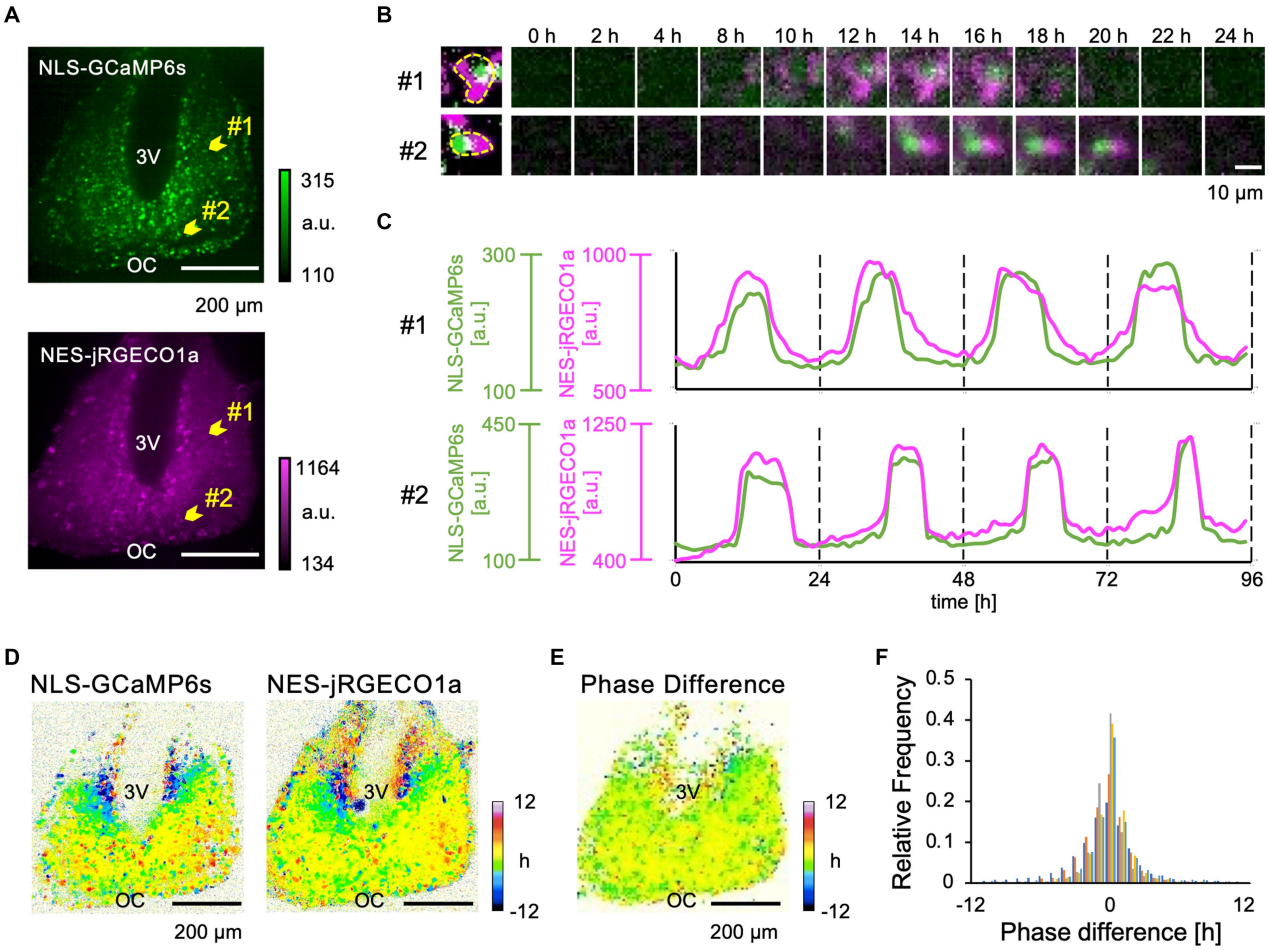
Effect of Ca^2+^ release inhibitors from the ER on the nuclear and cytosolic Ca^2+^ Rhythms. **(A–D)** Representative traces of the Ca^2+^ rhythms in the nucleus (green) and cytosol (magenta). The upper and lower traces in each panel show raw and 24-h detrended data (smoothed with a 3-h moving average), respectively. **(A)** Ryanodine receptor blocker, dantrolene (10 μM) (*n* = 5 slices). **(B)** Ryanodine receptor blocker, high concentration of ryanodine (100 μM) (*n* = 6 slices). **(C)** IP_3_ receptor blocker, Xestospongin C (10 μM) (*n* = 6 slices). **(D)** Sodium channel blocker, TTX (1 μM) (*n* = 5 slices). **(E–F)** Summary of blocker effects on the amplitude **(E)** and trough **(F)** of the nuclear (green) and cytosolic (magenta) Ca^2+^ rhythms. Data are expressed as percentage of the pretreatment value. One-sample *t*-test was employed to evaluate the blocker effects. *p < 0.05; **p < 0.01; ***p < 0.001. All data are expressed as mean ± SD.

To further investigate the contribution of Ca^2+^ release from the ER, we applied the IP_3_ receptor antagonist, Xestospongin C (10 μM), and statistically analyzed the effect on the Ca^2+^ rhythms. We found that neither the amplitude nor the trough level of both nuclear and cytosolic Ca^2+^ rhythms was affected (amplitude: 117% ± 12% in the nucleus, 110% ± 14% in the cytosol, trough: 104% ± 1% in the nucleus and 103% ± 2% in the cytosol) (*n* = 6 slices) (Figures 3C,E,F).

Next, we applied 1-μM TTX to block sodium-dependent action potential firing in SCN neurons. As previously reported (Ikeda et al., 2003; Enoki et al., 2012a), after the TTX application, the amplitudes of the Ca^2+^ rhythm in both the nucleus and cytosol significantly decreased in the entire SCN compared with the pretreat levels (69% ± 7% in the nucleus, 44% ± 4% in the cytosol) (*n* = 5 slices) (Figures 2D,E). The trough of the Ca^2+^ rhythm in the cytosol was also significantly reduced (98% ± 9%) but not in the nucleus (103% ± 1%) (Figure 2F).

Taken together, these results indicate that in-phasic Ca^2+^ rhythms in the nucleus and cytosol are mainly driven from the extracellular space rather than from internal Ca^2+^ stores in ER or nuclear envelope.

## 4 Discussion

### 4.1 Nuclear Ca^2+^ dynamics on the circadian scale

As is common in eukaryotes, the nucleoplasm is generally covered by a nuclear envelope that is contiguous with the ER, and the nuclear envelope contains nuclear pores (Devos et al., 2014). It has been reported that Ca^2+^ transporters, such as ryanodine and IP_3_ receptor, are expressed on the inner nuclear membrane (Bootman et al., 2009; Secondo et al., 2020). These structural features suggest that the nuclear envelope functions as a Ca^2+^ store and that nuclear Ca^2+^ dynamics are regulated independently of the cytosol. Indeed, nuclear and cytosolic Ca^2+^ dynamics have been previously reported in mouse SCN (Ikeda et al., 2003) and genetically modified tobacco seedlings (Wood et al., 2001), both of which show the Ca^2+^ rhythms in the cytosol but not in the nucleus. However, it is difficult to assume the functional independence of nuclear Ca^2+^ on a circadian time scale because of nuclear pore complexes. In this study, we observed the circadian Ca^2+^ rhythms in the cytosol and nucleus by expressing highly sensitive, single-molecule, genetically encoded fluorescent Ca^2+^ sensors, NLS-GCaMP6s and NES-jRGECO1a, in the SCN neurons. We concluded that the nuclear and cytosolic Ca^2+^ dynamics are synchronized on a circadian time scale.

### 4.2 Ionic origin of the Ca^2+^ rhythm

Among the three isoforms, the type 2 ryanodine receptor has been reported to be expressed in rodent SCN (Díaz-Muñoz et al., 1999). However, the involvement of the ryanodine receptor in Ca^2+^ rhythm regulation in the SCN neurons remains elusive. In the present study, we administered two types of ryanodine receptor blockers, dantrolene and ryanodine at a high concentration, and found that the amplitudes of Ca^2+^ rhythms were not significantly altered by either dantrolene or ryanodine, but the trough levels were slightly increased by a high dose of ryanodine. Dantrolene is known to decrease the open probability of ryanodine receptor type 2 in the presence of calmodulin (Oo et al., 2015), whereas ryanodine at a high concentration increases the open probability and decreases conductance (Thomas and Williams, 2012). These different pharmacological actions may account for the different effects on the trough levels of the Ca^2+^ rhythms.

Previous studies have demonstrated that the high dose of ryanodine reduced the amplitude of Ca^2+^ rhythms (Ikeda et al., 2003) and dantrolene decreased the Ca^2+^ levels at both the peak and trough phases in the SCN neurons of tissue cultures (Plante et al., 2021), whereas another study showed no significant effect on Ca^2+^ rhythms in dissociated SCN neurons (Noguchi et al., 2017). There have also been reports of Ca^2+^ level measurement when drugs were applied at a specific timing (CT 4–8), but the results were inconsistent; Ca^2+^ levels have been shown to increase, decrease, or remain unchanged, strongly correlating with the baseline Ca^2+^ levels measured at day and night in SCN slices (Aguilar-Roblero et al., 2016). The reason for these conflicting results is unknown, but it may be due to different experimental conditions (e.g., dynamic range and sensitivity of Ca^2+^ probe, gene transfection methods, timing of pharmacological administration) and/or heterogeneity of SCN neurons (Wen et al., 2020).

Regarding the contribution of IP_3_ receptors on Ca^2+^ rhythms in the SCN, it has been reported that IP_3_ receptors are involved in photoentrainment to the SCN (Hamada et al., 1999). Furthermore, because the Gq-mediated plasticity of the SCN network has been reported (Brancaccio et al., 2013), IP_3_-mediated Ca^2+^ release may be involved in light-induced resetting or synchronization of the SCN. In the present study, using an IP_3_ inhibitor, we found that the IP_3_ receptor is involved neither in the amplitude nor trough level of Ca^2+^ rhythms. Taken together, we conclude that neither ryanodine nor IP_3_ receptor-mediated Ca^2+^ release had a major contribution to the autonomous Ca^2+^ rhythms in SCN neurons.

A recent study demonstrated that the knockdown of the leucine zipper-EF-hand-containing transmembrane protein 1 (LETM1), a mitochondrial Ca^2+^/H^+^ exchanger, dampened the cytosolic Ca^2+^ rhythms in the SCN (Morioka et al., 2022) and that mitochondria Ca^2+^ content rhythmically oscillates in SCN astrocytes and U-2 OS cells (Scrima et al., 1867; Burkeen et al., 2011). Therefore, the mitochondria may function as an ion source of Ca^2+^ rhythms.

Cytosolic circadian Ca^2+^ rhythms have been reported in cultured SCN from neonate mice (Colwell, 2000; Ikeda et al., 2003; Enoki et al., 2012a, 2017a,b; Brancaccio et al., 2013; Noguchi et al., 2017) as well as in adult mouse SCN (Aguilar-Roblero et al., 2016; Jones et al., 2018). Since the development of the SCN has been reported (Myung et al., 2021), we can not exclude the possibility that the properties of the nuclear circadian Ca^2+^ rhythms are altered during development.

### 4.3 Function of nuclear Ca^2+^ signaling

Ca^2+^ is a molecule critical for the three essential properties of the circadian clock: autonomous oscillation, entrainment, and temperature compensation (Cavieres-Lepe and Ewer, 2021; Kon et al., 2021). The present study suggests that nuclear and cytosolic Ca^2+^ rhythms may regulate these circadian properties via Ca^2+^-dependent signaling *in situ*.

It has been reported that CaMKII and PKCα (protein kinase Cα), Ca^2+^-dependent kinases, are expressed in the SCN (Van der Zee and Bult, 1995) and are candidate molecules that can be directly activated by nuclear Ca^2+^. In general, these protein kinases are thought to translocate from the cytosol to the nucleus upon Ca^2+^ elevation; however, some isoforms have been reported in the nucleus of mammalian cells (Robles et al., 2010; Mishra et al., 2011). CaMKIV, CBP/p300 and Downstream regulatory element antagonist modulator might be other candidates for nuclear Ca^2+^-dependent kinases, although their subcellular expression in SCN neurons has not yet been investigated.

The current view of the TTFL model is based on the nuclear translocation of clock proteins (Mohawk et al., 2012; Takahashi, 2017); however, a recent live-cell imaging of endogenous clock proteins shows that clock proteins (e.g., BMAL and CRY1) are predominantly present in the nuclear compartment (Gabriel et al., 2021), challenging the view of circadian gating of nuclear import. It may be plausible that cytoplasmic Ca^2+^ translocates to the nucleus and regulates transcription of clock genes *in situ*.

This study highlights the important but long overlooked function of nuclear Ca^2+^, which may directly activate Ca^2+^-dependent kinases and regulate the transcriptional regulation of clock genes. The key molecules that could be activated by nuclear Ca^2+^ signaling remain to be identified in the SCN neurons, but this could be an important target for the Ca^2+^ signaling of circadian functions in future studies.

## Data availability statement

The raw data supporting the conclusions of this article will be made available by the authors, without undue reservation.

## Ethics statement

The animal study was approved by National Institute for Physiological Sciences. The study was conducted in accordance with the local legislation and institutional requirements.

## Author contributions

SH: Conceptualization, Data curation, Formal analysis, Investigation, Writing – original draft. KK: Methodology, Resources, Writing – review & editing. TN: Conceptualization, Funding acquisition, Project administration, Writing – review & editing. RE: Conceptualization, Funding acquisition, Investigation, Methodology, Project administration, Resources, Supervision, Writing – original draft.
