# Supplementary material for: In-phasic cytosolic-nuclear Ca2+ rhythms in suprachiasmatic nucleus neurons
Source: Front Neurosci. 2023 Dec 20;17:1323565. doi: 10.3389/fnins.2023.1323565 (PMC10765503; doi:10.3389/fnins.2023.1323565)
Supplement: Supplementary file 1 [file Presentation_1.pdf]

Supplementary Material

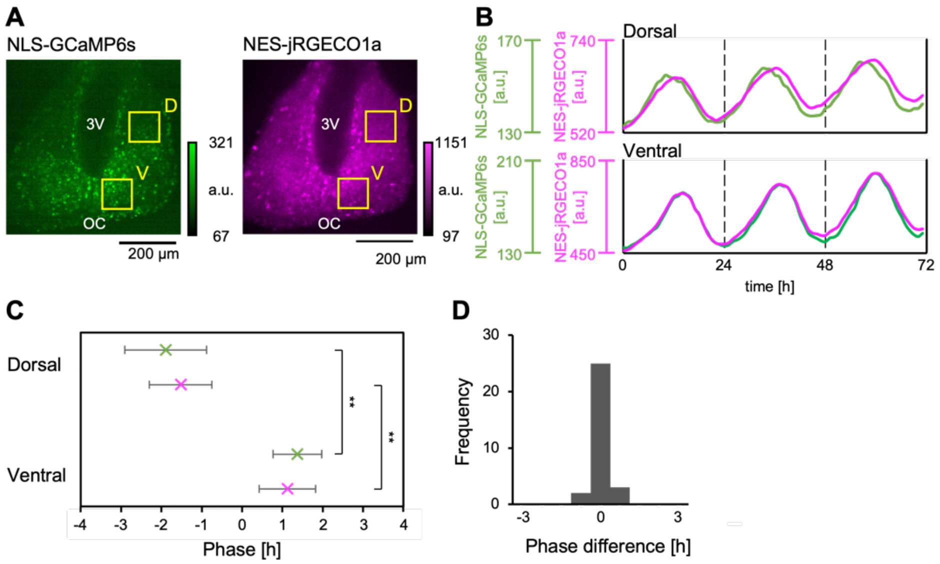

Figure S1. Cell-Based Analysis of the Nuclear and Cytosolic Ca^{2+} Rhythms

(A) SCN slices expressing NLS-GCaMP6s (left) and NES-jRGECO1a (right). ROIs (100 × 100-µm yellow squares) were positioned in the dorsal and ventral SCN subregions. 3V, third ventricle; OC, optic chiasm. **(B)** Regional traces of Ca^{2+} rhythms in the nucleus (green) and cytosol (magenta) in the ROIs in **(A)**. **(C)** Regional acrophase of Ca^{2+} rhythms in the nucleus (green) and cytosol (magenta) in the dorsal and ventral SCN. Mean acrophase of the entire SCN region normalized to zero. * * $p < 0.01$. Data are expressed as mean ± SD (n = 5 slices). **(D)** Histogram of phase differences in individual SCN neurons (n = 30).
